# Utilization of lightweight ceramic aggregates based on waste materials in the production of lightweight polymer concrete as a component of sustainable architecture

**DOI:** 10.1038/s41598-024-81290-5

**Published:** 2024-11-26

**Authors:** Jakub Smoleń, Klaudiusz Fross, Krzysztof Groń, Kaja Orzechowska, Krzysztof Stępień, Grzegorz Junak, Mateusz Kozioł, Sebastian Pawlak, Tomasz Pawlik, Roxana Fross

**Affiliations:** 1 Faculty of Materials Science, Silesian University of Technology, Krasińskiego 8, Katowice, 40-019 Poland; 2 Scientific and Didactic Laboratory of Nanotechnology and Materials Technologies, Faculty of Mechanical Engineering, Silesian University of Technology, Towarowa 7A, Gliwice, 44-100 Poland; 3 Faculty of Architecture, Silesian University of Technology, ul. Akademicka 2A, Gliwice, 44-100 Poland

**Keywords:** Architecture, Epoxy polymer concrete, Waste material, Lightweight ceramic aggregate, Lightweight concrete, Physical, Mechanical properties, Engineering, Civil engineering, Materials science

## Abstract

In this study, a novel lightweight epoxy polymer concrete (PC) was developed with lightweight ceramic aggregates based on waste materials, which can be applied in construction materials. For the purposes of this study, lightweight ceramic aggregates based on waste materials were produced and used as fillers in the production of epoxy polymer concretes. Two aggregate fractions were used in the study: 4–8 mm and 8–16 mm. The physical properties of non-infiltrated and infiltrated granules were compared, which clearly demonstrated that infiltration is beneficial, as the penetration of liquid resin into deep pores increases the interfacial surface area. Next, using the infiltrated granules, a series of polymer concretes were prepared and tested for compressive strength, flexural strength, open porosity, water absorption, apparent density, and thermal diffusivity. The highest mechanical properties were achieved for samples containing only a fine fraction with aggregates with a diameter of 4–8 mm, 99.68 MPa compression, and 18.71 MPa flexural strength. Thermal diffusivity measurements were obtained for heat transfer comparison between the developed polymer concrete and traditional concrete. The results showed that the thermal diffusivity value for the polymer concrete was equal to 2.33  ×10^-7^ m^2^/s, which was nearly half of traditional concrete. The investigated material was considered to be frost-resistant because of its low water absorption (0.36%). It was proven that the utilization of lightweight ceramic aggregates based on waste materials was reasonable and increased the mechanical properties of the polymer concrete alongside the overloading environment by processing wastes that are difficult to reuse.

## Introduction

The development of lightweight structural polymer concretes is an important area in the construction and production of building materials. Reducing the density of construction materials relieves the structure, reduces materials consumption and reduces the costs of transporting. The reduction of the concrete’s mass is possible in two ways: the introduction of air into the concrete matrix or the aggregate^1–2^. According to Newman and Owens^3^, lightweight concretes have an over-dry density (the bulk density after drying for 24 h in the air at 105 °C) with a range of 300 to 2000 kg/m^3^, compressive strength of 1 ‒ 60 MPa and thermal conductivity of 0.2 to 1.0 W/mK. For comparison, traditional concretes have an over-dry density of 2000–2500 kg/m^3^, compressive strength of 15 ‒ 100 MPa, and thermal conductivity of 1.6–1.9 W/mK.

The addition of lightweight ceramic aggregates (with bulk density reduced by about 1/3 to 1/2 compared to traditional heavy stone aggregates) into the concrete structure results in the desired reduction in density. The EN 13,055 standard provides key information on aggregate density, classifying all aggregates with a density below 2000 kg/m³ or a bulk density below 1200 kg/m³ as lightweight aggregates. The mechanical strength of concrete is related to the strength of the lightweight aggregate. The use of weaker aggregates requires the use of a stronger matrix (which increases the cement content) to maintain the compressive strength value. Improving the bonding of the matrix with the aggregate ensures the effective utilization of the properties of the matrix^4^. The nature of the cracking of concretes is determined by the tensile strength test, which differs between traditional concrete and lightweight concrete. The fracture path of traditional aggregates runs around the particles, while for lightweight aggregates, due to the reduced mechanical strength, the fracture path goes through the particles. Due to the favorable combination of low density and mechanical strength, it is possible to obtain lightweight concretes with significantly higher specific strength than traditional concretes^5–7^. Additionally, the presence of higher tensile strength of lightweight aggregates and lower modulus elasticity generates lightweight concretes with enhanced impact resistance than traditional concretes^8–10^. Concretes containing light aggregates in the structure are characterized by good resistance to thawing/freezing cycles^11^, reduced abrasion resistance^12^, higher water absorption^13^, lower resistance to carbonation^14^, lower thermal expansion, and lower thermal conductivity than traditional concretes^15–16^. Low thermal conductivity caused by the internal porous structure of lightweight concrete results in a good thermal and acoustic insulator^17–18^.

Lightweight ceramic aggregates (LCA), also called expanded clay aggregates^19^, are porous ceramic materials formed by high-temperature decomposition of selected mass components, which emit gases during decomposition. The emitted bubbles expand the structure of the ceramic, generating closed and open pores. The greater the volume of gases formed, the greater the porosity of the material, and this directly affects the lowering of the density. The production process of light ceramic aggregates occurs in a rotary kiln at a temperature of 900–1300 °C^20–22^. LCA is used in construction, especially in areas that require reduced self-load of the structure, as well as in gardening as drainage and filtration of the root zone of plants. Due to its porosity, expanded clay has favorable thermal and acoustic insulation properties^23–19^. Lightweight ceramic aggregates often consist of waste materials, including coal shale^24^, fly ash^25^, organic compounds^26–28^, plastics^29^, and others^30–32^. In the work of Adhikar et al., a broad review of research works focused on lightweight self-compacting concretes was conducted, where several types of lightweight natural and sustainable aggregates were described. They noted that it is possible to produce concretes with densities below 1000 kg/m^3^ with appropriate parameters^33^. The literature results allow us to understand that mechanical strength, density and water absorption are characteristic features of individual lightweight ceramic aggregates and determine the performance properties of concrete. The most commonly used types of lightweight ceramic aggregates include mainly: expanded clay^34,35^, pumice^36^, scoria^37^, waste rubber^38^, expanded shale^39^, perlite^40^, and many others.

Obtaining a composite structure containing the porous/foam structure as the filler positively influences the mechanical properties, heat resistance, wear resistance, thermal insulation, etc^41–42^. The basic problems occurring during infiltration include, among others, the selection of optimal process parameters, taking into account time, pressure, and temperature^43–44^. Infiltration of lightweight ceramic aggregates in the entire volume is considered impossible when there are closed pores filled with gas inside the material. However, open pores and through pores can be successfully filled with liquid phase, e.g., molten metal or polymer resin. The combination of the infiltration of open porosities on the surface of the aggregates and the preservation of empty pores inside may simultaneously increase the mechanical strength of the aggregate (and, as a result, an increase in the strength of concrete), increase the thermal insulation, acoustic insulation, and the overall reduction in the weight of the structure^45–46^.

Among the numerous types of concrete, polymer concretes are of great importance due to their potential properties and applications. In polymer concretes, the cement binder is replaced by a polymer, most often a resin. Compared to traditional concrete, it has various advantages, primarily: significantly shorter cross-linking time, higher strength properties (compressive strength, tensile strength), chemical resistance, abrasion resistance, lower water absorption, frost resistance, and resistance to freeze-thaw cycles^47–49^. The biggest limitation in the applicability of polymer concretes is the high unit price resulting from the price of the polymer (resin) and the equipment^50^. The cost-effectiveness of using polymer concrete instead of traditional cement-based concrete is closely related to the area of application. Most often, polymer concrete is used for the production of installations, resistance to chemical agents, construction projects (e.g., construction of bridges or pavements), production of marble artifacts (e.g., sculptures, kitchen countertops), repair of damage to traditional concretes, production of prefabricated products (e.g., road drains), waterproofing of buildings, and other areas^51–53^. In recent years, many researchers have focused on the development of new materials based on epoxy resin containing waste materials as fillers in terms of environmental protection^54–55^.

The development of modern architecture focuses not only on the implementation of current architectural concepts and setting future trends, but also attention is paid to modern architecture using innovative materials that meet specific structural requirements, but also contribute to the development of sustainable building practices. Creating new materials provides architects with solutions to reduce the carbon footprint of projects while ensuring that properties are maintained or improved^56,57^. It is desirable that the materials used in modern architecture and construction combine many beneficial features. materials combining many properties. An example of a hybrid material may be, a roofing board with many features combined in one element: structure, insulation, roofing, solar panel, power source for the heat pump^58^.

Herein, we examine the use of lightweight ceramic aggregates in the production of lightweight polymer concrete. The designed material combines the advantages of using polymer concrete and lightweight ceramic aggregates. The generated materials are expected to exhibit high resistance to chemicals, frost resistance, low water absorption, high mechanical strength, abrasion resistance, thermal insulation, acoustic insulation, and high specific strength. Additionally, we produced lightweight ceramic aggregates based on problematic industrial waste, especially contaminated glass cullets and coal shales. Several studies have been carried out to demonstrate the high application potential in the production of construction and building materials.

## Experimental

**Fig. 1:**
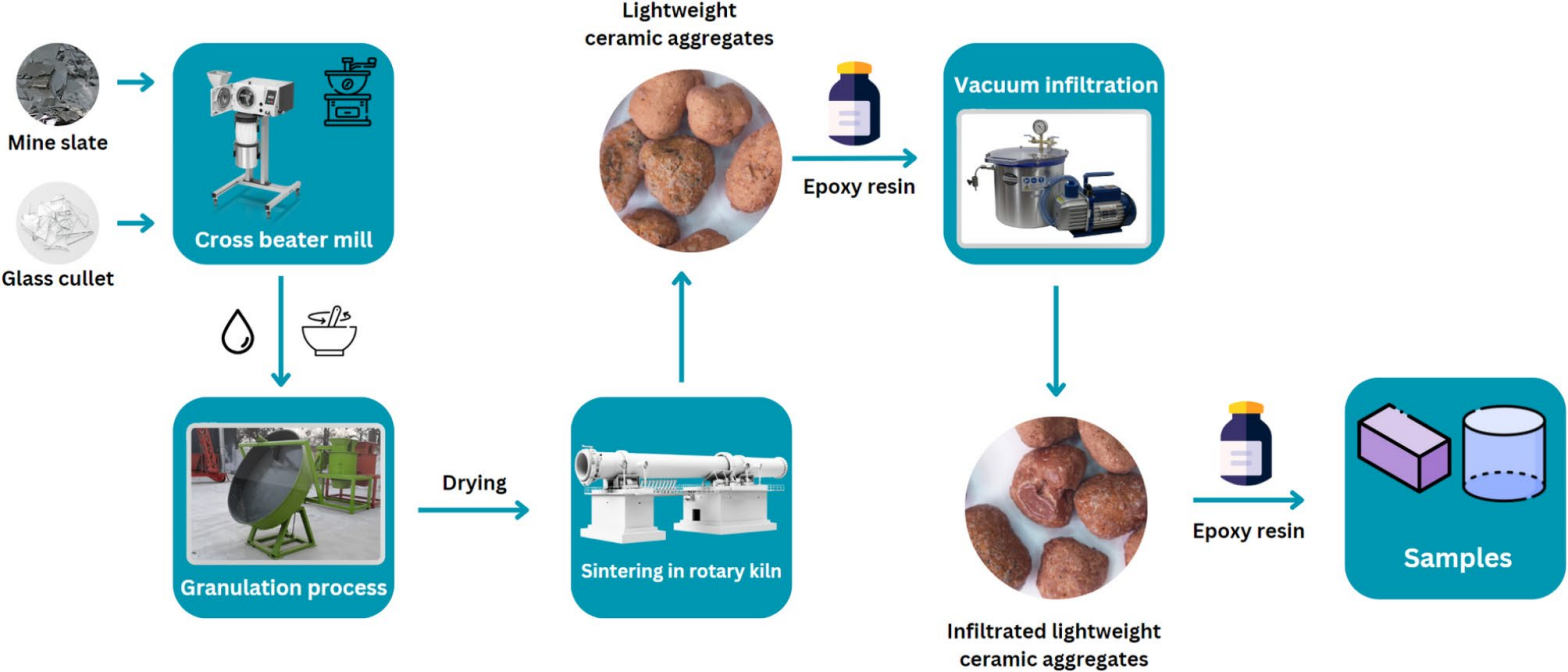
Diagram of the production of a polymer concrete filled with lightweight ceramic aggregates based on waste materials.

A lightweight structural polymer concrete was developed as follows: In the first stage, granules used to make polymer concrete were made from selected waste materials containing waste that is difficult to dispose of, such as waste glass from windshield car glass containing PVB and post-mining shales. The components were milled to a grain size less than 0.5 mm and then homogenization and granulation process. The dried granules were sintered in a rotary tube furnace at temperatures ranging from 900 to 950 °C for 15 to 30 min. In the second stage, the obtained granules were vacuum infiltrated with epoxy resin. Finally, these granules were used as aggregates to produce lightweight concrete, which was our main focus. A diagram representing the production of a polymer concrete filled with light ceramic aggregates based on waste materials is shown in Fig. 1.

### Materials

#### Components

Typical bisphenol A based epoxy resin LH288 and cycloaliphatic amine based hardener H505 (both from Havel Composites, Czech Republic) were used. The weight ratio of resin to hardener was 100:27. Resin and hardener with a gel time exceeding 30 min were employed, facilitating efficient processing. The resin was cross-linked at room temperature (approx. 21 °C) for 24 h and then post-cured at 70 °C for 12 h, according to the resin manufacturer’s recommendations. The post-curing process is not mandatory but is recommended for this resin as it allows for complete cross-linking of the epoxy structure.

**Fig. 2:**
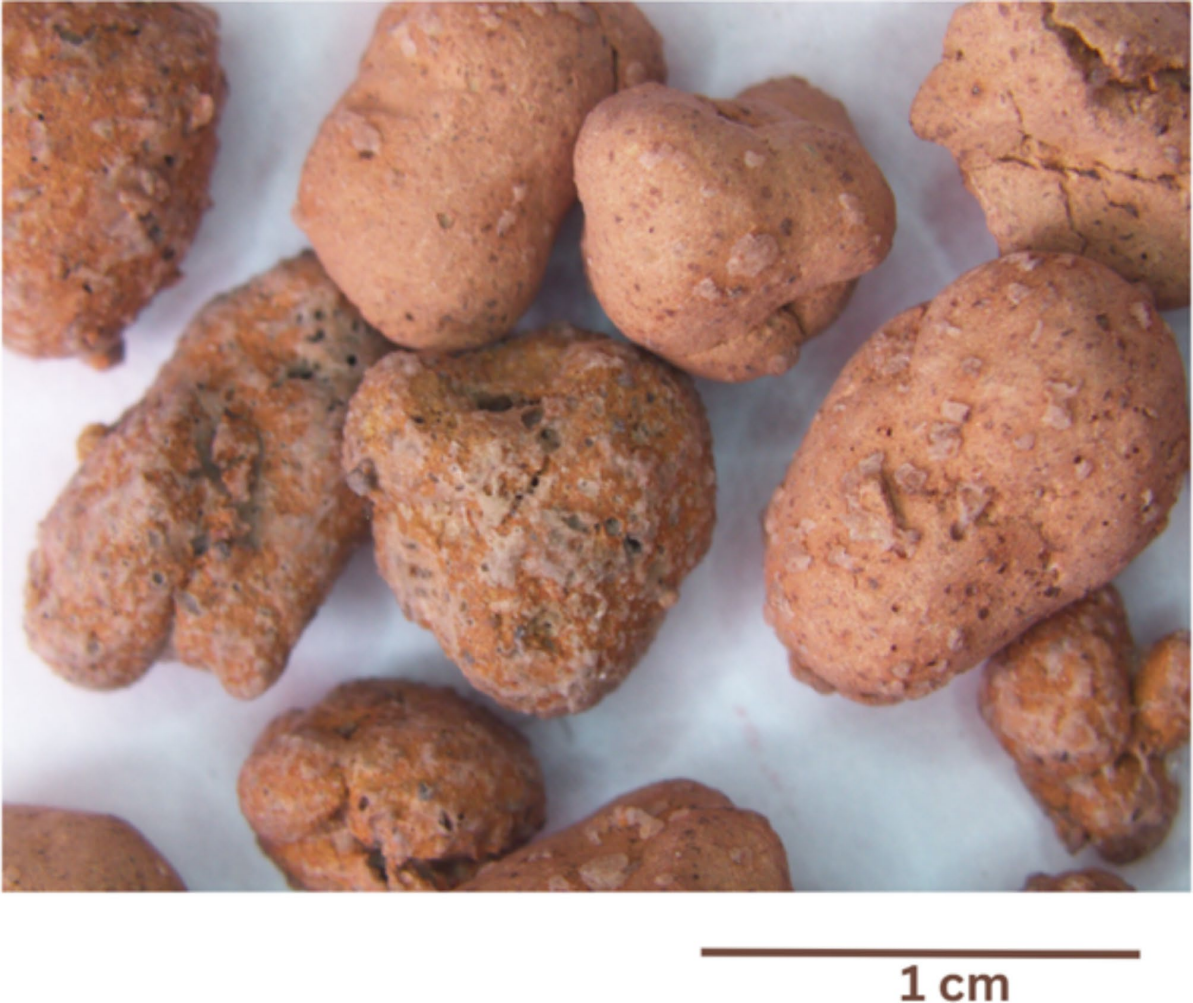
View of the sintered ceramic granules at ×6.7 magnification.

Lightweight ceramic aggregates based on waste materials were produced using a high-temperature sintering process. The raw materials were contaminated glass cullet from a car dismantling company and coal shales from Krupiński coal mine (Suszec, Poland). The raw materials were prepared by drying in a laboratory drier at 105 °C for 24 h. Then, the waste of the contaminated glass cullet and coal shales was milled in a Retsch SK300 (Retsch GmbH, Germany) cross-beater mill with a rotational speed of 3000 rpm using 0.5 mm separation sieves. The average grain size after milling was approx. 200 μm. The coal shale powder was mixed with the contaminated glass cullet powder in a weight ratio of 70:30. Distilled water was added to the homogeneous mixture of powders to obtain a moisture content of 10%, which favored the granulation process. The ceramic mass was placed in a mixer to compact the round granules. The obtained granules were dried at 105 °C for 24 h and sintered at 900–950 °C for 15–30 min. Gas emission during sintering was tested using a gravimetric dust meter EMIOTEST 2594 (E.M.I.O., Wrocław, Poland). The study showed limited, negative environmental impact caused by the emission of harmful compounds. Based on the measurements, it was found that dust, concentration of NO_2_, CO, CO_2_, SO_2_, aliphatic hydrocarbons up to C_12_ and hydrocarbons from C_2_ to C_6_ and formaldehydes are within the limits of environmental standards. The lightweight ceramic aggregates were divided into two size fractions: fine with aggregates of 4–8 mm and coarse with aggregates of 8–16 mm. Sintered ceramic granules are shown in Fig. 2.

#### Infiltration of the aggregates

The vacuum infiltration process was carried out in two stages. In the first stage, vacuum infiltration was used to fill open pores in the lightweight ceramic aggregates. The dry aggregates were placed in a liquid epoxy resin, and the system was placed inside a vacuum chamber. The vacuum infiltration process at a pressure of approx. 1 bar was carried out for 10 min (until no air bubbles were released). The aggregates were then separated from the residual liquid epoxy resin and allowed to cross-link. The second stage of the vacuum infiltration was carried out on composite granules after infiltration and cast into molds. A detailed description of the technology of infiltration of light ceramic aggregates is described in ref^59^.

#### Production of polymer concretes

Resin-infiltrated and cross-linked ceramic aggregates were mixed with liquid epoxy resin according to the compositions presented in Table 1.

**Table 1:** Volume content of components in the tested polymer concrete.

Sample name	LCA 4–8 mm	LCA 8–16 mm	Epoxy resin
PC1	60%	0%	40%
PC2	30%	30%	
PC3	0%	60%	

A series of traditional concrete samples were prepared to compare the results. Baumit B40 fast-hardening concrete (Baumit, Poland) was used and prepared in accordance with the manufacturer’s recommendations in a weight ratio of dry to wet ingredients of 100:10.

### Methods

#### Molding samples

Polymer concrete and traditional concrete were prepared by casting in molds. In order to perform the three-point bending test, samples with dimensions of 200 × 50 × 25 mm (length x width x height) were prepared, and for the compression test, cylindrical samples with a diameter of 50 mm and a height of 100 mm were prepared. The Archimedes test was carried out on samples with dimensions of 30 × 15 × 10 mm.

#### Testing mechanical properties

The compression test was carried out in accordance with the PN-EN ISO 604 standard on the MTS-810 testing machine at the strain rate of 10 mm/min. Flexural strength was determined in accordance with PN-EN 12390-5 standard on an INSTRON 4469 testing machine with a strain rate of 10 mm/min and a support spacing of 190 mm.

#### Determination of porosity, water absorption, and density

The Archimedes test was carried out to determine the open porosity, apparent density, and water absorption of the lightweight ceramic aggregates before and after the infiltration process and to determine these properties in the final polymer concrete and traditional concrete (reference material).

Open porosity was determined according to the formula (1):

1 $$\:{P}_{o}=\frac{{m}_{2}-{m}_{1}}{{m}_{2}-{m}_{3}}\cdot\:100\text{\%}$$

The apparent density was calculated according to the formula (2):

2 $$\:{d}_{p}=\frac{{m}_{1}}{{m}_{2}-{m}_{3}}\cdot\:{d}_{c}$$

Water absorption was determined according to the formula (3):

3 $$\:{A}_{b}=\frac{{m}_{2}-{m}_{1}}{{m}_{1}}\cdot\:100\text{\%}$$

where: m_1_ – dry weight of sample, m_2_ – saturated weight, m_3_ - suspended weight, d_c_ - liquid density.

#### Determination of the thermal diffusivity

In order to investigate the heat transfer performance of the considered polymer concrete material, thermal diffusivity was selected as the parameter to be determined due to the possibility of direct comparison with a concrete sample. Determination of thermal diffusivity was achieved using the conventional heat pulse method based on ASTM E1461 standard^60^. The experimental procedure was carried out using an in-house apparatus for transient thermography measurements, where a transverse thermal diffusivity value was obtained. As a thermal wave source, a 1.2 kW infrared radiator with a wavelength range of 2–10 μm was used. The back surface temperature increase was recorded using the infrared camera FLIR A325 (FLIR Systems, Inc., USA), using a frame rate of 15 Hz. More detailed information about the applied apparatus can be found in ref^61^. The duration of the heating was 4.0 s for both materials tested. Based on the recorded temperature variations versus time plots, the normalized temperature plots were created to determine the value of the half-time t_0.5_ (the time to reach half of the maximum temperature increase on the back surface of the sample). The values of t_0.5_, together with the thickness (L) of the sample, were used to determine the thermal diffusivity values based on the formula: 1.38L^2^/π^2^t_0.5_^62^.

## Results and discussion

### Characteristics of lightweight ceramic aggregates

**Fig. 3:**
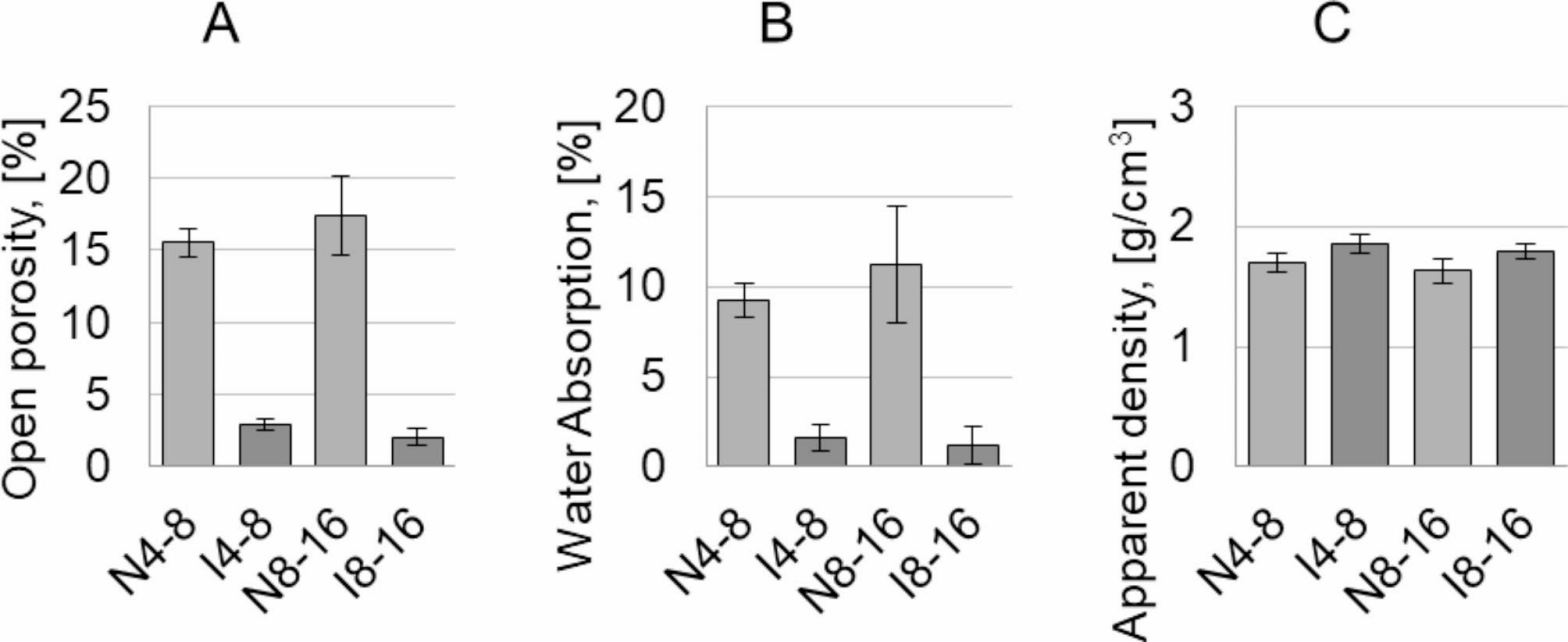
Archimedes test result for lightweight ceramic aggregates before infiltration and after infiltration process: (**A**)- open porosity, (**B**)- water absorption, (**C**)- apparent density.

The results of the Archimedes test are shown in Fig. 3. The Archimedes test showed that the aggregates had a high open porosity of approx. 15% before the infiltration process.

Infiltration of the aggregates with epoxy resin resulted in a 6-fold decrease of open porosity to approx. 2.5%. The reduction of the open porosity also decreased the water absorption. Initially, the water absorption value of the ceramic aggregates was approx. 10%. However, after filling the pores with epoxy resin, an 8-fold decrease to approx. 1.25% for both aggregate fractions was observed. The apparent density of the samples before infiltration was approx. 1.7 g/cm^3^. Filling the pores with resin resulted in a marginal increase in this value of around 0.2 g/cm^3^. The obtained lightweight ceramic aggregates are significantly lighter than traditional aggregates used in concretes, as quartz aggregates are characterized by a density of about 2.65 g/cm^3^, limestone aggregates - about 2.5 g/cm^3^, granite aggregates - about 2.7 g/cm^3^, basalt aggregates − 3.0 g/cm^3^.

### Characteristics of lightweight polymer and traditional concretes

**Fig. 4:**
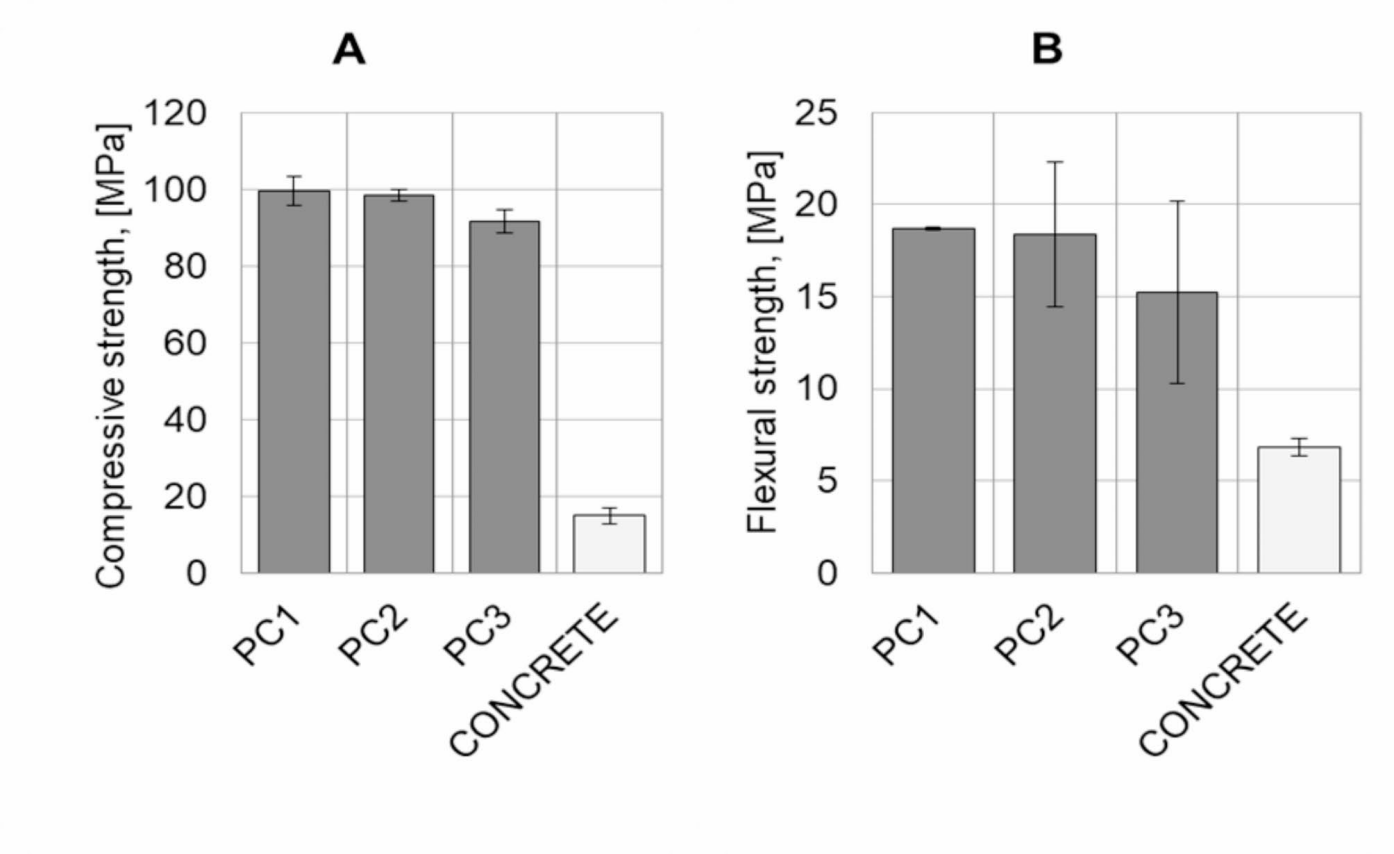
Compressive strength (**A**) and flexural strength (**B**) of the tested materials (14 days after production).

**Fig. 5:**
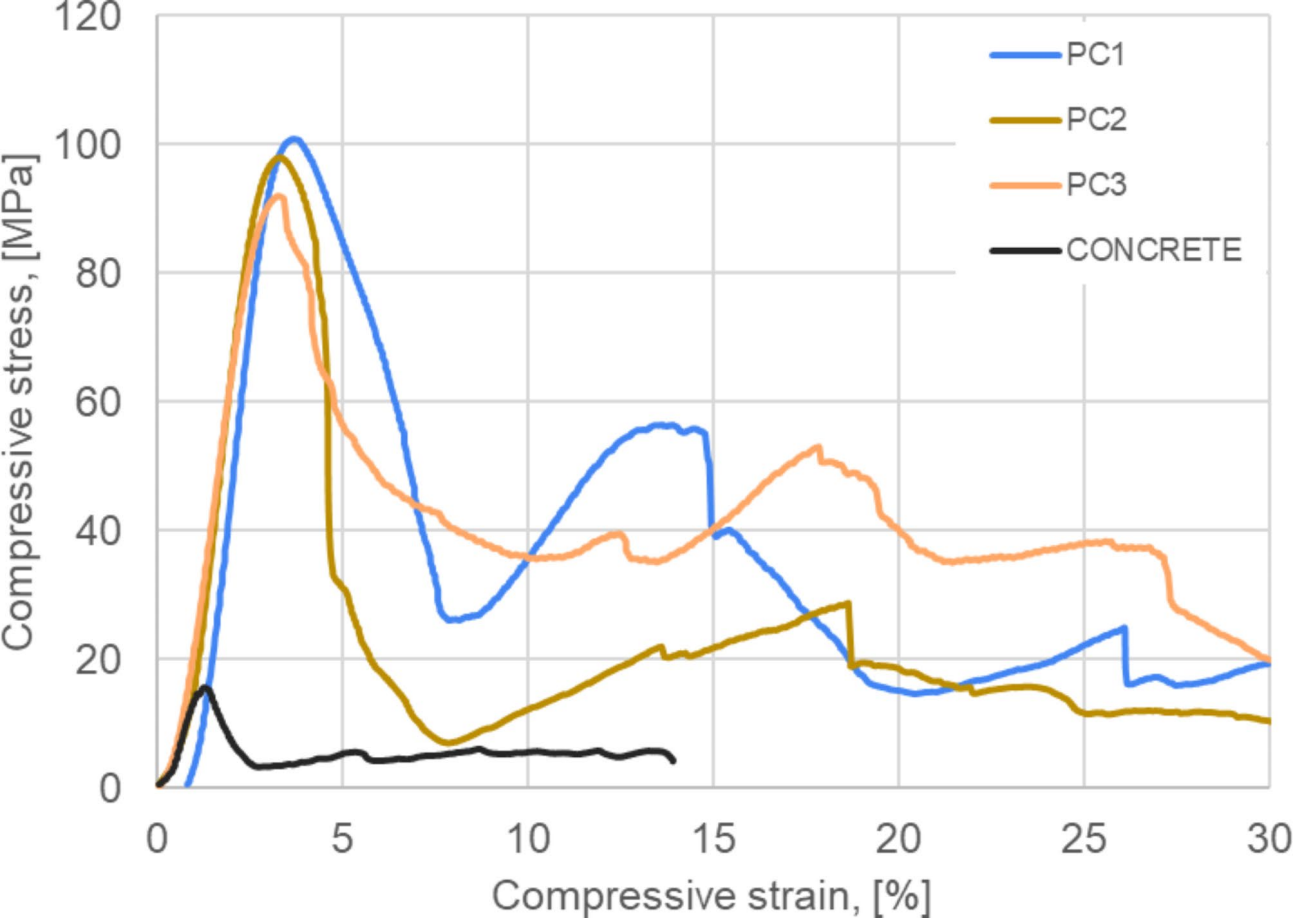
Representative static compression curves for the tested materials (14 days after production).

The results from the mechanical properties tests of the manufactured polymer concrete, as well as conventional concrete, are depicted in Fig. 4. Mechanical tests were carried out 14 days after production of polymer concretes and cement concretes. It can be seen that the manufactured polymer concrete samples exhibited much higher mechanical strength compared to concrete in both compressive and flexural strength. The value of the compressive strength of all polymer concrete samples was approx. 100 MPa, which, when tested with ordinary concrete, resulted in a 4-fold increase. The compressive strength graph shown in Fig. 5 revealed that the conventional concrete failed immediately, while the series for the polymer concretes showed a renewed but much smaller increase in strength after cracking. Hence, the material’s failure was not immediate, and the samples were still able to transmit compressive stresses of lower strength. Beneficial structural strengthening as a result of LCA infiltration was previously described in ref^48^. The resin penetrated only into the open, surface pores, while deeper, closed pores remained unfilled. The penetration of the resin into the empty spaces of the granules during the infiltration process allowed an increase in the interphase boundary, which ultimately had a positive effect on the mechanical properties of the concretes. It was observed that the large granules showed larger areas of pores, unfilled by the resin. The area of the halved unfilled pores was smaller in the finer aggregates. Such an effect directly impacts the mechanical strength as well as the deviation of results in the series containing the 8–16 mm fraction (samples PC2 and PC3). Compared to obtained polymer concrete samples with cement-based concrete containing lightweight aggregates obtained by Lo and Cui in ref^2^. , the produced polymer concrete showed a twice higher compressive strength.

**Fig. 6:**
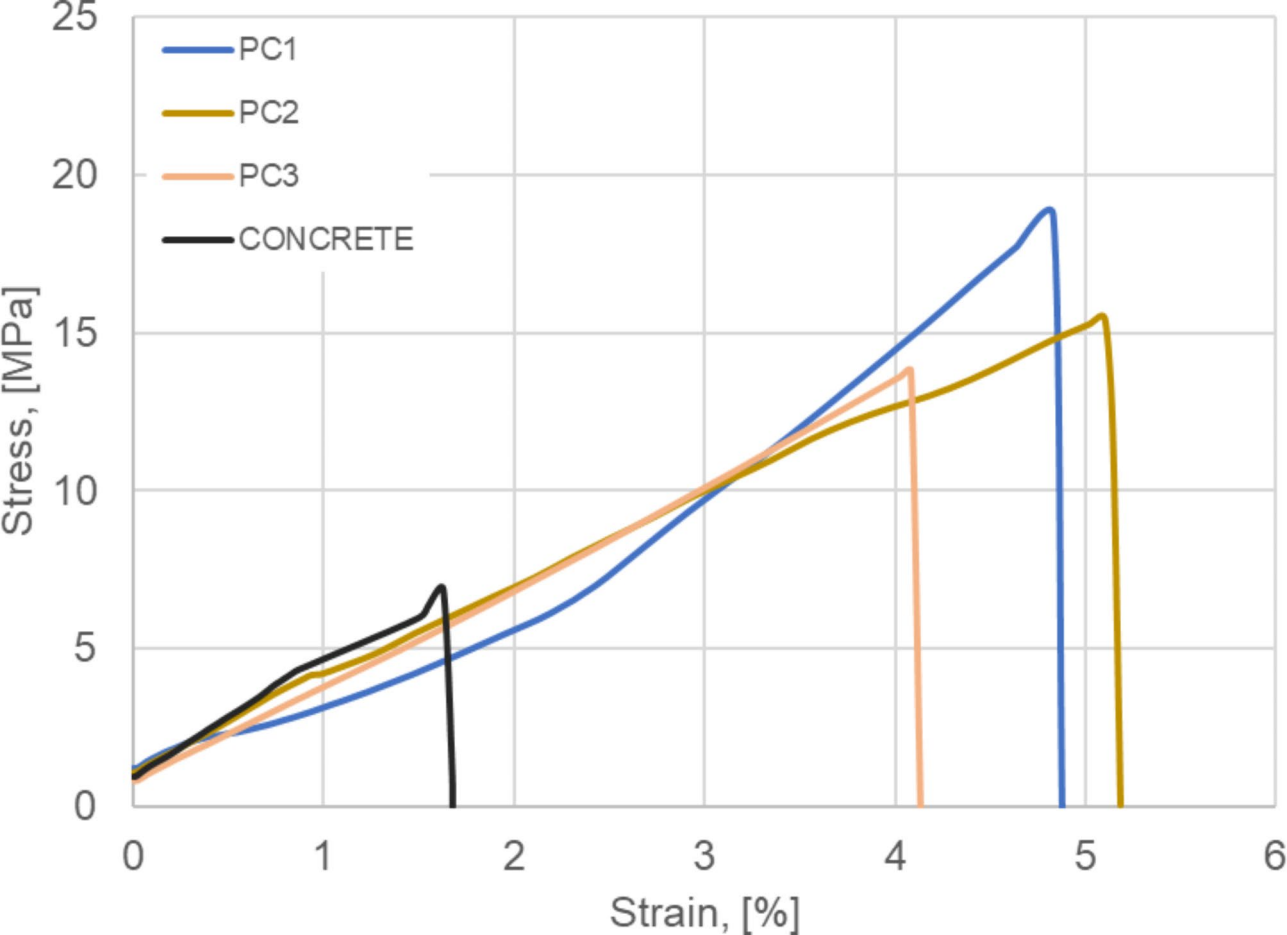
Representative static bending curves for the tested materials (14 days after production).

The three-point bending test showed that the polymer concrete samples had at least twice the flexural strength of the B40 concrete sample. The flexural strength of the polymer concrete samples ranged from 14 to 19 MPa, where, in comparison, B40 concrete was approx. 7 MPa. As shown in Fig. 6, the polymer concrete samples displayed a higher level of plasticity, achieving a strain of between 4.1% and 5.2%. The strain of conventional concrete during the test was approx. 1.7%. The highest obtained flexural strength value of the received material in comparison to concrete with the addition of a multiphase lightweight aggregate reported by J. Meng et al.^8^ was approx. 12 MPa higher.

**Fig. 7:**
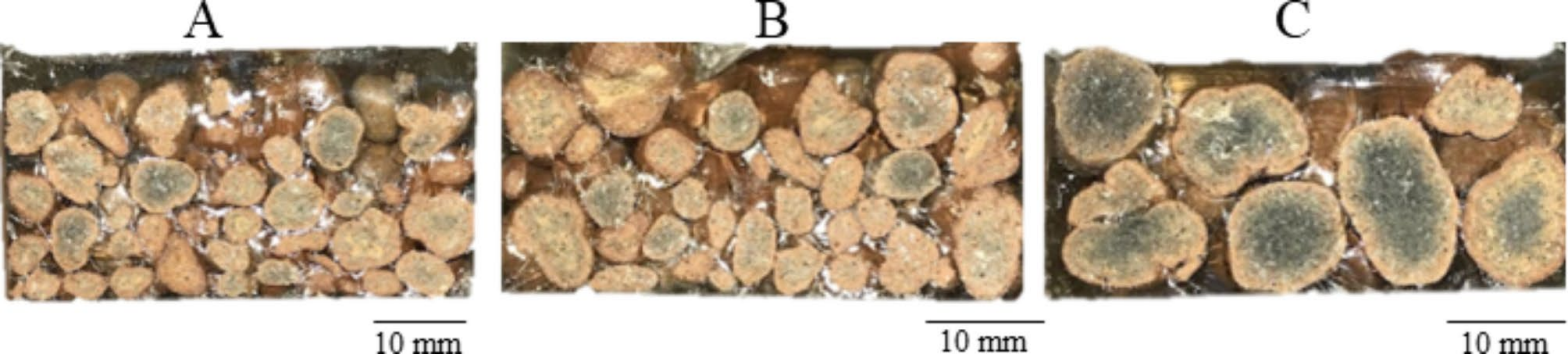
Fractures of samples after the three-point bending test. (**A**)- sample PC1, (**B**)- sample PC2, (**C**)- sample PC3.

**Fig. 8:**
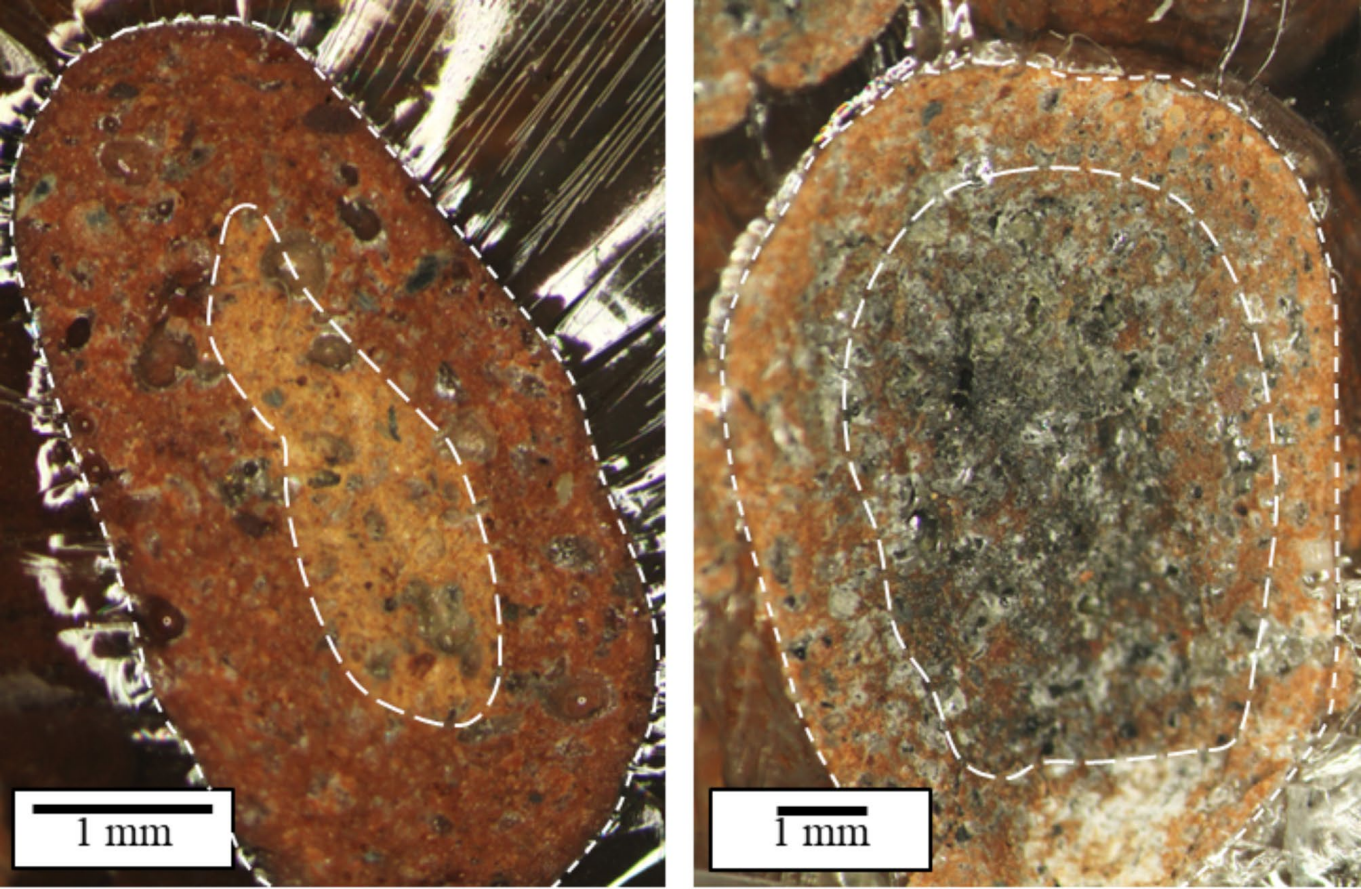
Granule infiltration zones, on the left is a small granule less than 8 mm, and on the right is a large granule above 8 mm (stereoscopic microscopy technique).

Based on the results of the mechanical tests and observations of fractures of the polymer concrete samples (Fig. 7), we found that the PC1 series, containing only granules with a diameter of 4–8 mm, had the smallest dispersion of values and was characterized by high homogeneity. Figure 8 shows the cross-section of the studied granules, where the depth of penetration of the resin into the granules is visible. The outer layer of the granules had a high resin content, which had infiltrated the open and through pores on the surface, while the inner layer (core) had a low content of filled pores. The zone of unfilled pores inside the granules was significantly larger for the large aggregates.

The arrangement of granules in the polymer concrete allowed for an even distribution of LCA in the volume, while large granules in the PC3 sample generated a large variation of mechanical properties. Additionally, between LCA and large, free spaces filled with epoxy resin were observed, which was unfavorable. Therefore, due to the advantageous results obtained for small granules (4–8 mm), further tests (Archimedes test and thermal diffusivity assessment) were carried out on the PC1 samples.

**Fig. 9:**
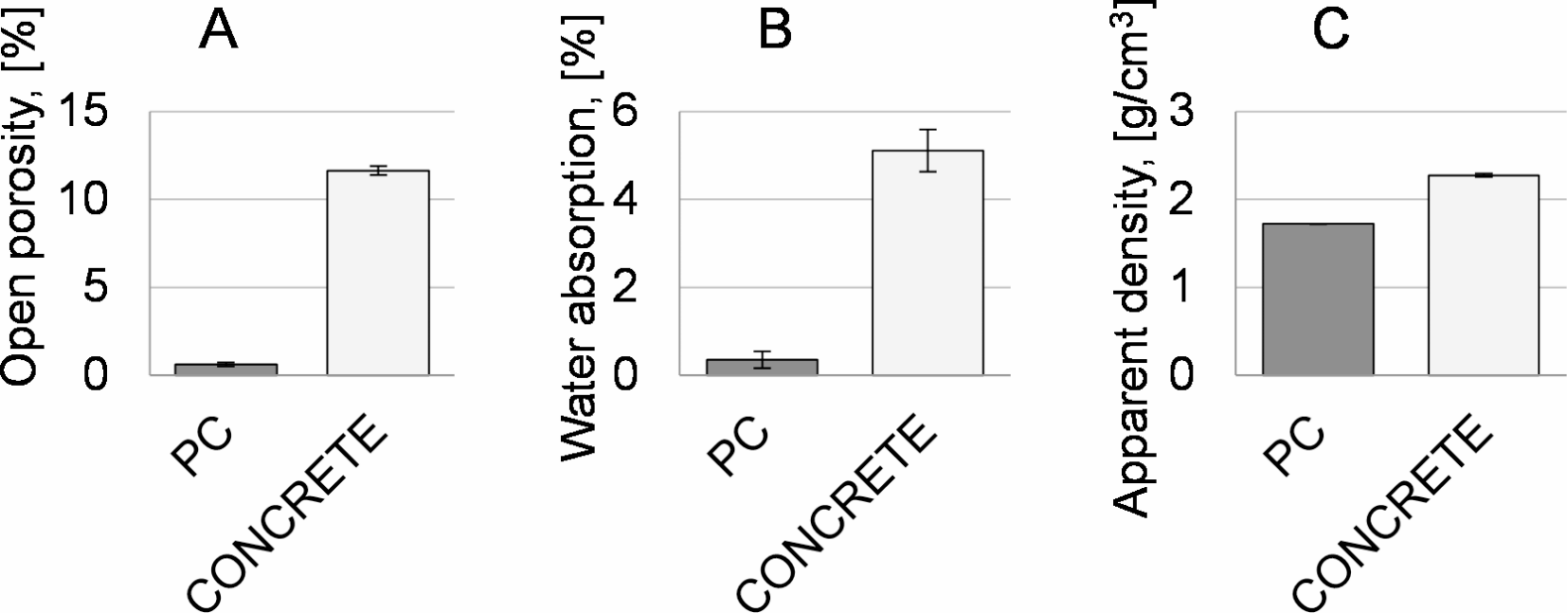
Archimedes test results for reference concretes and lightweight polymer concretes (PC1): (**A**)- open porosity, (**B**)- water absorption, (**C**)- apparent density (14 days after production).

A direct comparison of the Archimedes test results (open porosity, water absorption, apparent density) between the polymer concrete and B40 concrete is shown in Fig. 9. The results highlighted the superiority of the polymer concrete samples over conventional concrete in each of these aspects. The open porosity of the polymer concrete material was approx. 0.7%, compared to cement concrete had a 17-fold lower value of 12%. As a result of lower open porosity, the polymer concrete displayed a low level of water absorption with a rate of approx. 0.3%. The water absorption value of the sample made of conventional concrete was approx. 5%. The apparent density value determined by the Archimedes test showed that the polymer concrete and conventional concrete were 1.8 g/cm^3^ and 2.2 g/cm^3^, respectively. The polymer concrete showed a higher relation of strength to apparent density than the B40 concrete.

### Heat transfer performance

**Fig. 10:**
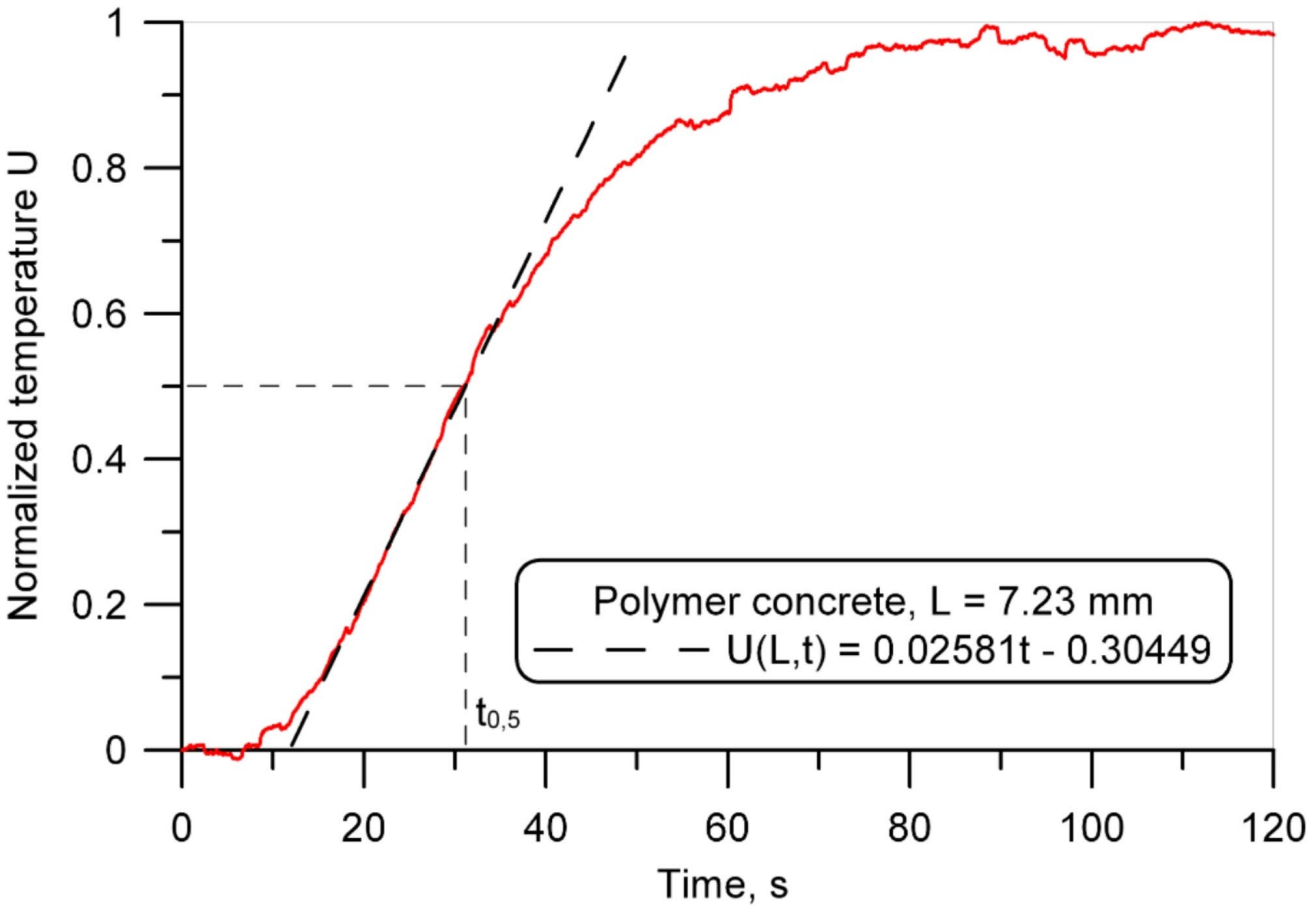
Normalized temperature increase on the back surface of the polymer concrete sample (sample PC1) (14 days after production).

**Fig. 11:**
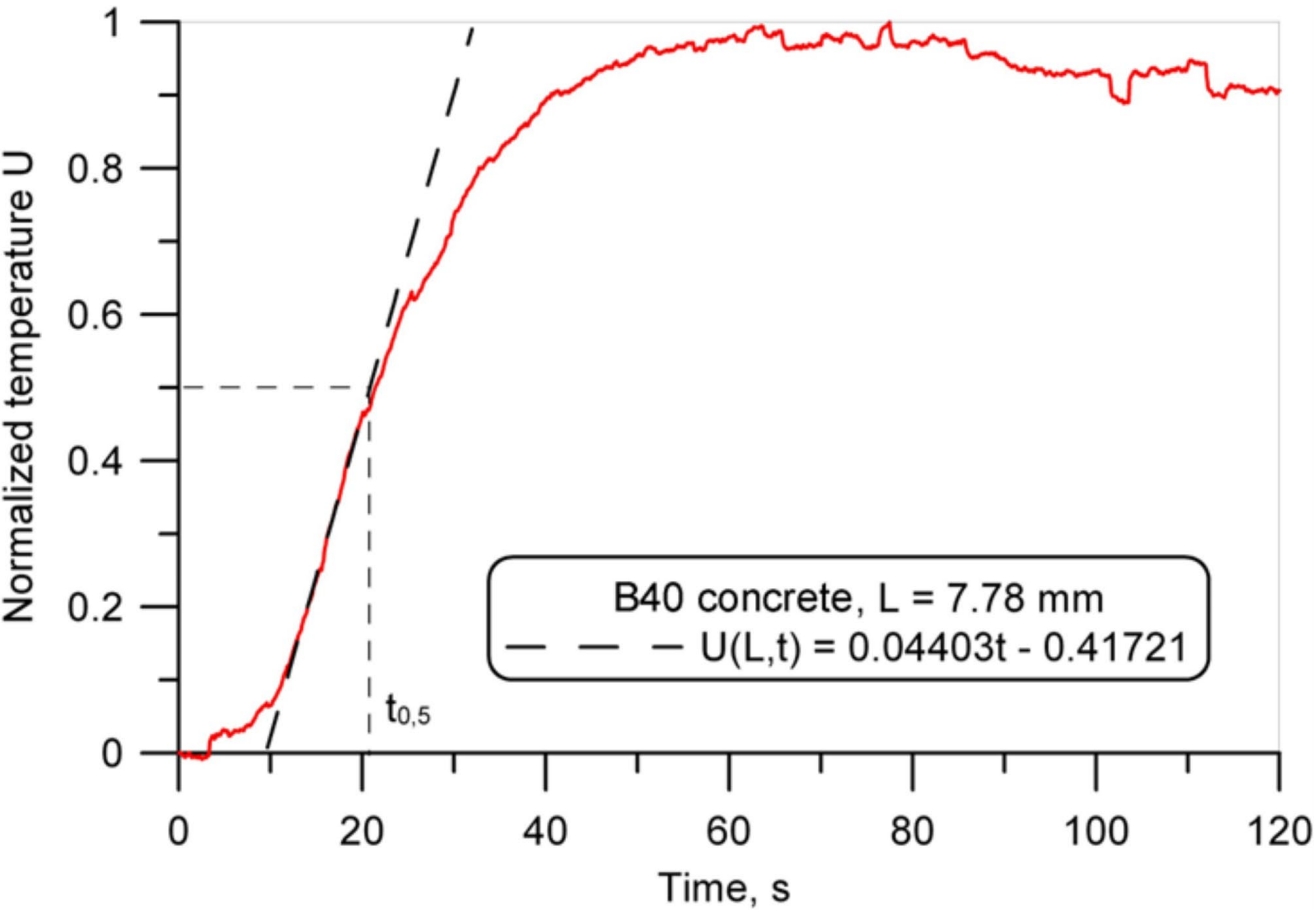
Normalized temperature increase on the back surface of the B40 concrete sample (14 days after production).

The measurements of the thermal diffusivity were carried out on square samples (100 mm x 100 mm), and the thickness values are listed in Table 2. An example of the dimensionless temperature-time plots on the back surface obtained for the polymer concrete and conventional concrete samples is shown in Figs. 10 and 11. The comparison of the temperature rise rate on the back surface of the samples (Figs. 8 and 9) revealed that it was much greater for the concrete sample and confirmed the higher thermal insulating behavior of the polymer concrete. The obtained thermal diffusivity value of 2.33 × 10^− 7^ m^2^/s for polymer concrete was almost twice lower as for the reference conventional concrete (Table 2).

**Table 2:** Results of the thermal diffusivity measurements for the polymer concrete and concrete samples obtained using the heat pulse method based on ASTM E1461 standard (14 days after production).

Material	Thickness (L), mm	Half time (t_0.5_), s	Thermal diffusivity, m^2^/s
Polymer concrete (sample PC1)	7.23	31.17	2.33 × 10^-7^
B40 concrete	7.78	20.83	4.03 × 10^-7^

The obtained properties of the tested polymer concretes showed evident superiority over the traditional concrete. However, the main critical issue was the economic efficiency of the potential application of the materials. Hence, the tested polymer concretes could be a serious competitor for traditional concrete, if they may be used in some special applications, demanding especially high strength, chemical resistance, and long-term reliability.

The produced lightweight polymer concrete is characterized by a set of beneficial properties combined in one construction material. This hybrid building material can be used with great success in modern architecture, where the environmental footprint of buildings, energy efficiency, energy saving and pro-ecological friendliness are becoming increasingly important. The material produced in these research is characterized by a combination of high mechanical strength and low weight, and additionally provides thermal insulation, acoustic insulation and high corrosion resistance. This combination of favorable properties means that it can replace traditional concrete in places such as ceilings, roofs or foundations. The developed material is characterized by low surface porosity and low water absorption, which allows us to conclude that it is a frost-resistant material, which reduces the risk of damage to architectural elements as a result of changing weather conditions. Moreover, high chemical and corrosion resistance means that the appearance of buildings and other architectural elements is preserved for a longer time than in the case of conventional cement concrete elements.

**Fig. 12:**
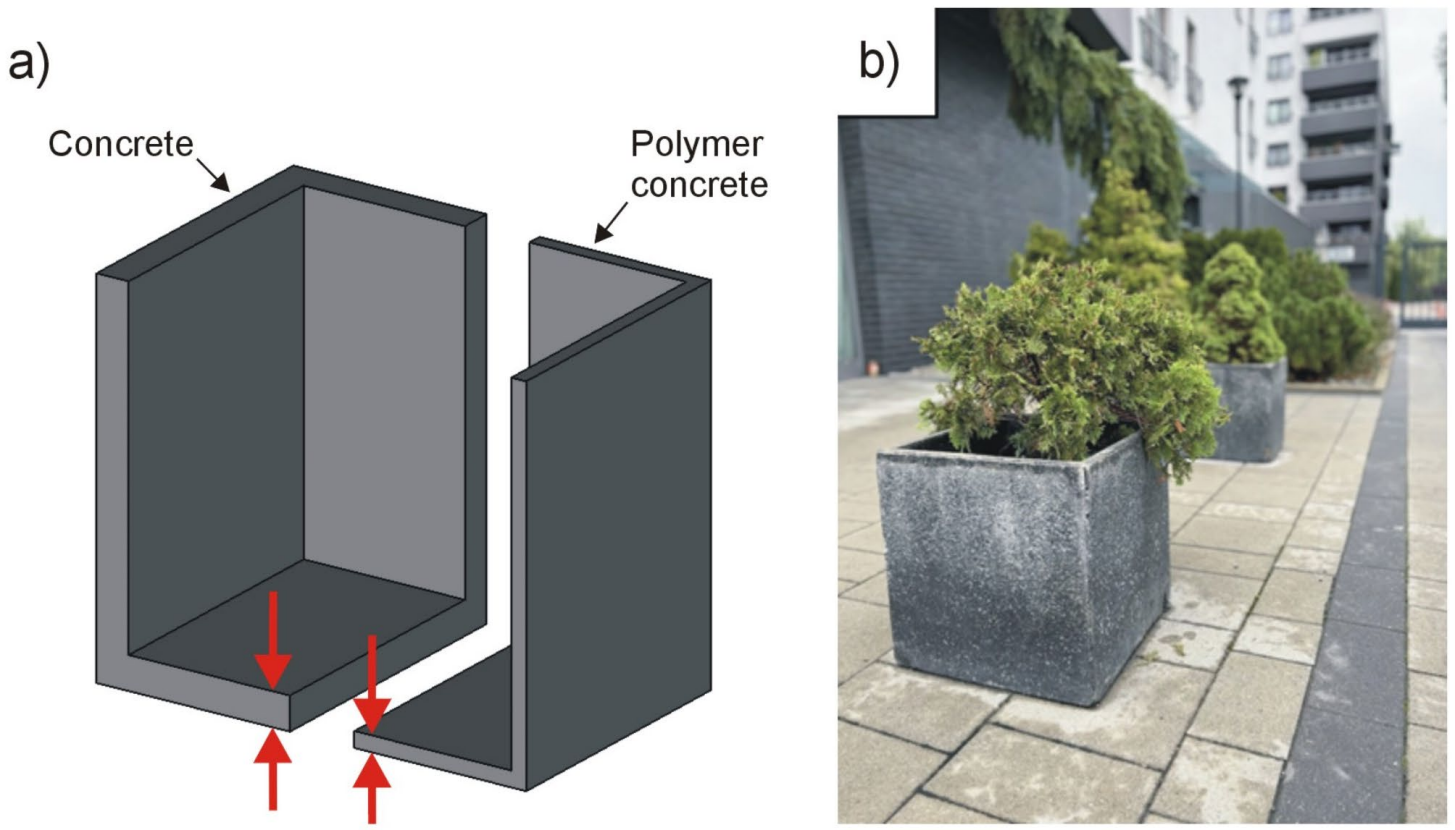
Application of polymer concrete in landscape architecture on the example of a garden pot: (**a**) 3D model, (**b**) photograph of garden pot.

**Fig. 13:**
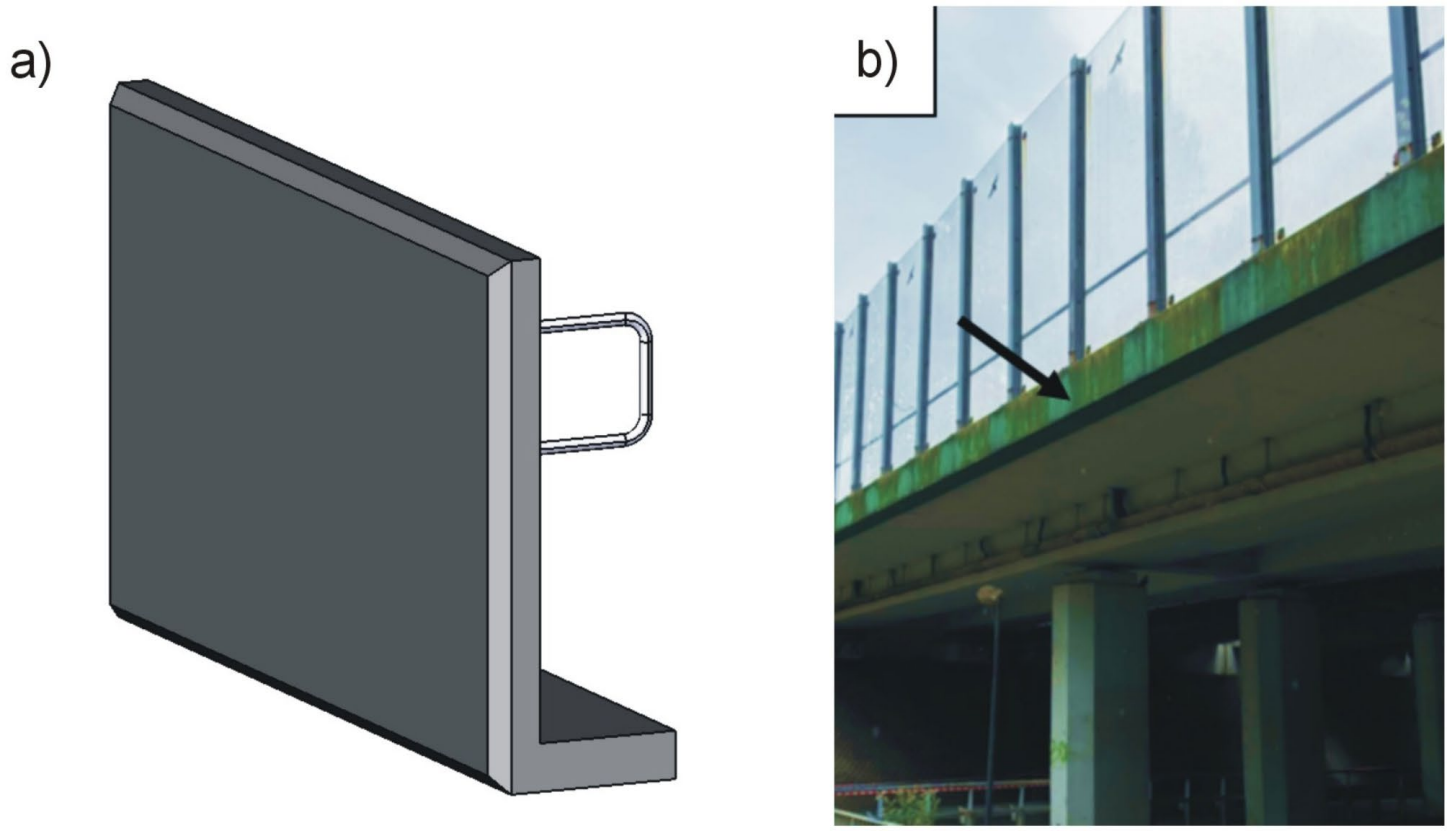
Application of polymer concrete in landscape architecture on the example of a cornice board: (**a**) 3D model, (**b**) photograph of road bridge.

In recent years a significant increase in the use of polymer concrete in architecture has been observed. An example of the use of polymer concrete in landscape architecture are garden pots (Fig. 12). A comparative overview of wall thickness has been presented for traditional concrete and polymer concrete planters. The results demonstrate a significant reduction in wall thickness for the polymer concrete planter, attributed to the higher strength of polymer concrete. Pots made of polymer concrete are characterized by thin walls that improve the aesthetics of the object, are mechanically strong and resistant to environmental conditions, and are also lighter than typical pots made of cement concrete with thick walls. Additionally, polymer concrete mixed with lightweight ceramic aggregates offers improved thermal insulation, which is beneficial for plant roots. This insulation protects plants from overheating during summer and from freezing during the winter. A good example of combining the functionality of objects made of polymer concrete with aesthetics and safety are cornices on bridges, viaducts and other road infrastructure objects (Fig. 13). Cornices made of polymer concrete provide an aesthetic finish to the edges of bridges, are lightweight, making them easier to install, and additionally ensure safety in use. The key task of the cornices is to protect the users from being hit by falling crumbled pieces of concrete. The polymer concrete cornice, as more durable than the rest of the construction made of classic concrete, simply stops the crumbled pieces from falling down. The cornices also ensure a proper water drainage. The use of polymer concretes in bridge construction is possible. However, it is important to remember about certain limitations in the applicability of polymer materials. One of the potential limitations is the high temperature of use, which can lead to creep of polymer materials. It is therefore important to select materials rationally, paying particular attention to the glass transition temperature (T_g_) of the resin, which determines the maximum operating temperature of this material without damage. In this study, an epoxy resin was used, the glass transition temperature of which is higher than 80 C, which ensures the durability of the material under high temperature conditions of use.

In conclusion, this study fills a certain research gap by introducing a novel approach to the production of lightweight polymer concretes. The use of lightweight ceramic aggregates made from waste materials such as car windows containing PVB foil and coal shale, and the use of vacuum infiltration to enhance resin bonding, is an important innovation. This method results in significant improvements in mechanical properties, thermal insulation and frost resistance compared to traditional concrete. The practical applications of the proposed improvements are very promising for architecture, providing improved thermal properties, durability in the service environment, as well as aesthetic appeal of architectural elements. Combining sustainability with high performance, this study sets a new benchmark for lightweight concrete materials in architectural applications.

## Conclusions

Our study examined the production potential of lightweight polymer concretes with the use of porous ceramic aggregates. Ceramic aggregates were produced on the basis of waste materials. The obtained test results for the tested polymer concretes were compared with typical cement-based concrete. The following results were determined:

- The obtained polymer concrete belongs to the class of lightweight concretes due to its density being below 2000 km/m^3^,
- Vacuum infiltration of porous aggregates allows for deep penetration by the resin, which increases the surface area of the interface and has a beneficial effect on mechanical strengthening,
- The compressive strength of the polymer concrete is about 100 MPa, and the bending strength is from 14 to 19 MPa, which significantly exceeds the properties of traditional concrete, 14 days after production,
- The best mechanical properties are characterized by polymer concrete composed only of fine aggregates (4–8 mm), where the material shows good homogeneity and small variation in mechanical properties,
- The use of porous ceramic aggregates increases the thermal insulation of the material almost twice compared to traditional concrete (thermal diffusivity value for polymer concrete is equal to 2.33 × 10^− 7^ m^2^/s),
- The production of lightweight polymer concrete based on porous aggregates is beneficial because the material obtained is lighter than traditional concrete, has better thermal insulation, is more mechanically durable, and has better frost resistance and water absorption. However, economic calculations reveal that their applications may be limited to specific cases demanding special performance.
- Based on the analysis of the state of knowledge, it is predicted that polymer concretes show greater durability and unchanging appearance compared to traditional cement concretes. Such material behavior is beneficial and allows to maintain the appearance of architectural elements for a longer period of time. The importance of polymer concretes for architectural sciences will be a subject of further research.

## Data Availability

The datasets used and/or analysed during the current study available from the corresponding author on reasonable request.
